# Overexpression of a fusion defensin gene from radish and fenugreek improves resistance against leaf spot diseases caused by Cercospora arachidicola and Phaeoisariopsis personata in peanut

**DOI:** 10.3906/biy-1902-25

**Published:** 2019-04-05

**Authors:** Madhu BALA, Thankappan RADHAKRISHNAN, Abhay KUMAR, Gyan Prakash MISHRA, Jentilal Ramjibhai DOBRAIA, Pulugurtha Bharadwaja KIRTI

**Affiliations:** 1 Department of Biotechnology, Directorate of Groundnut Research , Junagadh, Gujarat , India; 2 Department of Biotechnology, University of Hyderabad , Hyderabad, Andhra Pradesh , India

## Corrigendum

This corrigendum for “Overexpression of a fusion defensin gene from radish and fenugreek improves resistance against leaf spot diseases caused by Cercospora arachidicola and Phaeoisariopsis personata in peanut” (Turkish Journal of Biology 2016; 40 (1): 139-149; doi: 10.3906/biy-1412-46) is issued to replace a figure. This figure is of the amplification of the selectable marker used in the gene construct. While preparing Figure 2B, we inadvertently used a figure of the same gene amplification from another construct and hence duplicated this figure with that of Fig 3(B) of The Scientific World Journal (doi: 10.1155/2014/125967). We have prepared a new Figure 2B showing the PCR amplification from the construct referred to in this publication as a replacement, which is presented below. This correction does not in any way compromise the findings of the study in terms of the methodology, results, or interpretations drawn from the data therein. Any mention of this figure in the text should refer to this replaced figure.

**Figure 2:**
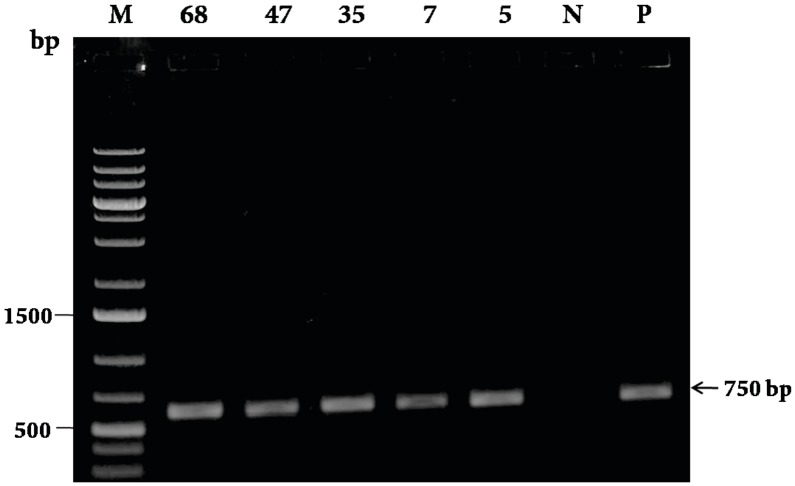
Preliminary confirmation of putative transgenic peanut plants by PCR amplification of nptII gene using gene specific primers. Lane M indicates 1-kb plus DNA ladder; Lanes 2–6 indicate five transgenic lines, namely DEF.68, 47, 35, 7, and 5 in the T0 generation showing the presence of the nptII gene; Lane N indicates negative control (nontransformed plant); Lane P indicates positive control (pRD- 400 vector with the nptII gene).

